# A Review of Studies on the Seasonal Variation of Indoor Radon-222 Concentration

**DOI:** 10.3389/or.2022.10570

**Published:** 2022-09-05

**Authors:** Guadie Degu Belete, Aragaw Msganaw Shiferaw

**Affiliations:** Physics Department, College of Natural and Computational Sciences, Assosa University, Assosa, Ethiopia

**Keywords:** indoor radon, environmental factors, seasonal variation, lung cancer, building

## Abstract

Due to their electrostatic nature, radon decay products can attach to solid particles and aerosols in the air. Inhalation and ingestion are therefore the two main routes through which people are exposed to radon and its decay products. During the inhalation of these radioactive aerosols, deposition takes place in different regions of the human respiratory tract. The deposited aerosols carrying radon and its progeny undergo a continuous radioactive transformation and expose the lung to ionizing alpha radiation, which can destroy the sensitive cells in the lung, causing a mutation that turns cancerous. Radon which is a colorless, odorless, and tasteless radioactive noble gas is a major health concern and is the second leading cause of lung cancer. To address this, an indoor radon survey was conducted in many countries internationally, with results showing that indoor radon concentration has a seasonal variation. This is due to the fluctuation of environmental parameters and the geological nature of buildings. Its concentration was found to be maximum in the cool (winter) season and a minimum concentration was recorded in the warm (summer) season of the year.

## Introduction

Since the existence of life began, living things in the universe, including humans, animals, and plants, have been exposed to natural radiation (1). Our world is full of radioactivity, with over 60 radionuclides found in nature (2). These radionuclides are the source of radioactivity and emit nuclear radiation which has become a part of our daily lives. Radiation is present everywhere and human beings are directly or indirectly exposed to radiation continuously. This radiation comes from different natural and human-made sources. The ionizing radiation originates from soils, water, building materials, air, water, mining areas, and cosmic origins, etc (2). Every day, we ingest or inhale nuclides in the air we breathe, in the food we eat, and in the water we drink. Therefore we have to investigate whether the natural radioactive level of the environment where human beings live is suitable for healthy living.

Human populations can be exposed to manmade and natural radiation sources. The natural radiation surrounding life on earth can either be terrestrial or extraterrestrial (cosmic) in origin. Terrestrial radiation includes the ionizing radiation arising from radionuclides in the earth’s environment, originating from the soil, rocks, construction materials, water, air, and mining areas, and cosmic rays are high energy radiations that enter the earth’s atmosphere from outer space (3). Radioactive elements such as ^238^U, ^232^Th, and their decay products, ^226^Ra, ^222^Rn, as well as ^40^K are major sources of radiation of natural origin (4). Ionizing radiations such as α, β, and γ radiations are emitted out from different terrestrial materials from which soil is a major source of natural radioactivity, as the main source for the migration and transfer of radionuclides into the surrounding environment (5). Different studies have been undertaken all over the world to determine the activity concentration of these radioactive elements, which are the main sources of natural radiation in the soil through which human beings experience direct contamination (6). The magnitude of these natural exposures depends on geographical location and some human activities (7).

Human beings can also be exposed to man-made radiation sources in addition to natural radiation sources. Radiation has different applications in various sectors such as medicine, biology, industry, agriculture, and electric power generation. During their application, humans can be exposed to the radiation emitting from different radioactive sources and exposed to different radiation-induced diseases (8). Man-made sources, known as artificial radionuclides, include medical radiation sources (x- rays and radioactive isotopes that are used in medicine for diagnosis and therapy), consumer products: [Such as static eliminators (containing polonium-210), smoke detectors (containing americium-241), cardiac pacemakers (containing plutonium-238), fertilizers (containing isotopes potassium, from uranium and thorium decay series), and tobacco products (containing polonium-210 and lead-210)], as well as atmospheric testing of nuclear weapons (radiations released during the testing of nuclear weapons and nuclear power generation). More than 80% of human exposure comes from natural radioactivity from different sources (9), and the rest is contributed by man-made radiation sources.

Radon (Rn), which is a radioactive decay product of one of the members of the uranium decay series called radium (Ra), is a radioactive, colorless, odorless, and tasteless noble gas, which makes it difficult to detect without special equipment. There are three known radon isotopes; Radon (^222^Rn) which has a half-life of 3.82 days, Thoron (^220^Rn) which has a half-life of 55.8 s, and Actinon (^219^Rn) which has a half-life of 3.98 s ^222^Rn, ^220^Rn, and ^219^Rn are found in the decay chain of the three uranium isotopes of ^238^U, ^236^U, and ^235^U, respectively (9, 10, 11). Uranium can be found in soil, rock, building materials, groundwater, and mining areas (12). Even though soil is the major source of radon, different building materials such as cement, rock, concrete, marble, paints, and gypsum contain uranium and radium (13). Radon is the leading source of ionizing radiation received by humans. It contributes around 55% of the environmental background radiation dose identified as a health hazard for mankind (13, 14). It is found in variable concentrations from location to location depending on the geological nature of that particular place (11).

**FIGURE 1:**
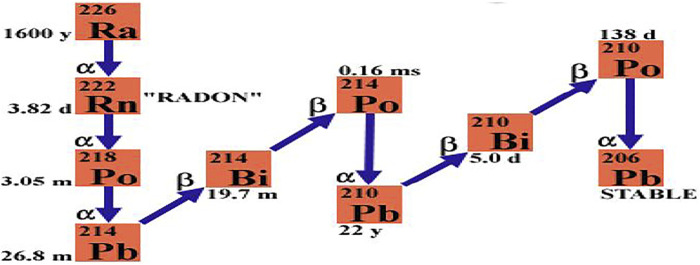
Radioactive decay chain of radon.

Radon is radioactive nuclei, which means it is unstable and hence undergoes a continuous radioactive transformation and forms several short-lived radioactive decay products called radon progenies. Polonium (^218^Po), lead (^214^Pb), bismuth (^214^Bi), and polonium (^214^Po) are successive radon decay products. Under each radioactive transformation alpha radiation, beta radiation, or sometimes gamma radiation is emitted (11). Over 90% of the radiation dose from radon is contributed by the two alpha radiation emit decay products called polonium (^218^Po) and lead (^214^Pb) (15). Figure 1 presents radon found from radium and its decay products are unstable and undergo radioactive decay by emitting ionizing alpha and beta particles which can cause great damage to human tissues.

### Indoor Radon and Its Health Effect

The earth’s atmosphere is full of gas with liquid and solid particles. Particles in the air are known as aerosols, which have various sizes, shapes, and chemical compositions. Mostly radius and diameter of aerosols can be used to define their size. Due to their size and differences in shape, they have different settling velocities and diffusion coefficients.

**FIGURE 2:**
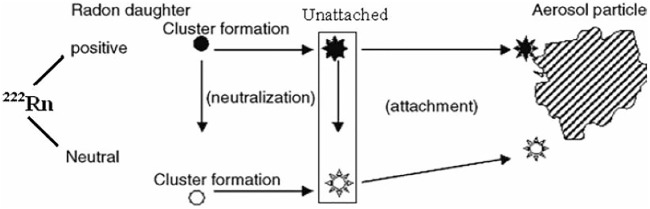
Aerosol formation of radon decay products.

When radon is exhaled into the earth’s environment from different sources such as soil, rock, building materials, groundwater, and mining areas through diffusion and emanation, its daughters become attached to the mono-dispersed and poly-dispersed aerosols due to their electrostatics characteristics (16). In these radioactive decay products, electrons are stripped from the parent atom by its recoil, and decay products are formed as positive ions. These ions can attract liquid and even solid materials from their surroundings, thus forming clusters of atoms or particles in the submicron region ranging from 0.0005 to 10 μm (17). Figure 2 presents the mechanisms of radioactive aerosol formation. Depending on the amount of aerosol concentration of the surrounding environment and humidity of the surrounding environment, around 80% of the radon decay progenies will be attached to the aerosols in the air that we take in.

Inhalation and ingestion are the two main routes by which human beings are exposed to radon and its decay products. After the inhalation and ingestion of aerosols carrying radon take place, it releases energetic alpha radiation with some associated gamma radiation that can damage the sensitive cells of the lung and stomach and induce cancer. Radon is a noble gas, it is non-reactive and is exhaled after being breathed in, but its decay products combined with other particles in the air such as dust or aerosols will be deposited on the surface of different regions of the respiratory system. Two of its decay products, Polonium (^218^Po) and lead (^214^Pb) are significantly hazardous. The deposited radioactive aerosols continued to undergo a successive radioactive spontaneous transformation. Under each decay, energetic alpha radiation is bombarded with the vital molecules in the lung cells causing a DNA structure break which causes mutations that turn into lung cancer (18).

Based on several investigations in Europe, North America, and China there is a direct relation between indoor radon concentration and inducing lung cancer. In these studies, radon was identified as the second leading cause of lung cancer after cigarettes (9). Different countries and organizations have dedicated activities to reducing radon health risks, as people need to take action when radon concentration exceeds the recommended safe limits. The World Health Organization (WHO) and Environmental Protection Agency (EPA) suggest that homeowners take action when radon levels exceed 100 Bq/m^3^ and 148 Bq/m^3^, respectively (11, 15).

### Major Factors Affecting Indoor Radon Concentration

Exposure to radon and its progeny is not limited to specific industries, for example, underground miners, and people working in indoor environments are also exposed to radon and its decay products. Uranium is a widely distributed constituent of the earth’s crust, typically in 2–4 parts per million, and in consequence, is found in most materials commonly used by the building industry. In the confined places of buildings and houses that do not allow air exchange radon gas can accumulate, and diffuse from building materials and soils, meaning that the people living and working there can easily inhale radon and its decay products (19) and it can accumulate to a harmful level. Different housing and environmental factors such as the types of construction materials of the building, the soil composition around the house, design of the house, construction, and level of ventilation of the house are major factors that determine the amount of indoor radon (^222^Rn) concentration (20).

The probability of inducing lung cancer in an occupied room is increased when the indoor radon concentration is high. Hence, different studies have been conducted concerning radon and its decay products. To understand the distribution of radon concentration in dwellings, different indoor radon surveys have been performed in different countries of the world. The results of these surveys indicate that the amount of indoor radon concentration has a strong variation with time. In general, one can study this in terms of two types of variations: daily and seasonal variation. In the diurnal context, the amount of indoor radon concentration is found to be maximum during the night and early morning, while the minimum concentration is recorded during the day (9). To manage the health hazards of radon and its progeny, one needs to study the seasonal variation of indoor radon concentration.

The results of different papers show that the concentration of radon and its decay products in dwellings varies from season to season. Meteorological parameters, geology, building materials, building construction type, and the degree of ventilation in closed environments are among the most important factors affecting radon in indoor air (21). The fluctuation of weather or environmental parameters such as temperature, pressure, humidity, ventilation condition, and wind speed and direction plays a role in the seasonal variation of indoor radon concentration (22).

Variations in the concentration of radon in homes are related to seasonal change. This is because climate change can cause different effects in terms of the environment or indoor air (22). The windows and doors of buildings during the winter months tend to be closed for longer periods of time due to rain, snow, or ice, which results in a lower ventilation rate in the room, and hence the accumulation of indoor radon tends to rise and can build to harmful levels. During the summer months, people open doors and windows, which increases the ventilation of the house (23). Ventilation rate and radon concentration have an inverse relation. Ventilation rate is key to reducing indoor radon concentration (22) as improvements in ventilation systems normally change radon concentration by less than 50 percent (24). Indoor radon concentration in winter therefore tends to be higher as compared to the other seasons of the year.

The structural formation of the building is also another factor in the variation of the concentration. The geology of different building materials such as cement, rock, concrete, marble, paints, and gypsum always contain uranium and radium (25). Buildings are made from rocks of different ages, origins, minerals, and chemical compositions. Heavy construction materials such as concrete and stone generally increase the thermal mass of the building, meaning there is an increase in the internal air temperature, which keeps the building warmer in summer (26). For this purpose, homeowners can apply different ventilation methods such as installing a radon pump system, opening windows, doors, and vents of the house (called natural ventilation), or using house pressurization *via* a fan. This makes the radon concentration seasonal, as it is low in warm (summer) and higher in cool (winter) seasons (16, 27).

## Result and Discussion

Different articles suggest that there is a relationship between the variation of indoor radon concentration and the construction style of a house. Some houses are constructed with a basement and others are constructed without a basement. The basement is an important construction element of a building. Radon concentrations in buildings with and without basements are different (28). Houses with a basement have greatly increased levels of radon concentration compared to those without a basement. Since radon enters homes from the ground, the presence of a basement is expected to be a determinant of high concentrations (29). Soil is most frequently the main source of radon in building air. The soil permeability of the basement is primarily responsible for the migration of radon into basements, which allows the gas to build up to harmful levels. In confined spaces such as the basements of houses and buildings where air exchange is not allowed, radon can accumulate to harmful levels. It is recommended that basements should not be used for residential purposes if the radon concentration is high.

The construction of the house plays a role in the variation of indoor radon concentration. The construction of houses in urban and rural areas is different due to economic, social, and environmental factors, as reflected in the type of buildings in urban and rural areas. In rural areas such as villages and hamlets, most houses are constructed from mud and local stones. These houses tend to have poor ventilation and are constructed with or without windows. The building materials of these houses, namely stone and soil, allow more radon to diffuse into the room due to the porosity of the materials used, which contributes to a high concentration of radon (25). The buildings in some rural areas of thwe world are smaller, older made from mud (30).

Wind speed and direction are other factors that affect the variation of indoor radon concentration as they affect the pressurization of a room and there is quite often a pressure difference between inside air and atmospheric air (28). In addition, the lifestyle or habits of the homeowners, in terms of shutting and opening doors and windows is another factor that influences levels of indoor radon concentration (22).

The age of a house determines the construction features of the house in terms of technologies and materials. Cracks and lack of continuity appear as construction materials age and increase radon inflow (28). Older homes have higher concentrations of radon because they typically have more cracks in flooring and the foundation and thus have a higher risk of contamination (22).

As has been observed from the data of different published papers, radon concentration is not constant with time. Its concentration shows variations day-to-day and season-to-season. To manage its adverse health effects, one needs to detect its seasonal concentration.

### Measurement Technique and Methodology

Different instruments can be applied to measure indoor radon concentrations from active and passive detectors. A passive integrated CR-39 alpha tract detector can be used to measure long-term radon-222 concentration.

In terms of methodology, measurement should ideally be performed four times a year, corresponding to the four seasons, to observe seasonal indoor radon concentrations. The detectors should be placed in the most frequently used parts of the house where the room occupants spent most of their time, such as bedrooms, sitting rooms, or kitchens, etc., To observe the effect of the types of building materials used, detectors should ideally be placed in houses made from different building materials with different designs at a height of 1 m from the floor and 0.5 m from the walls of the building (31, 32). Parallel to the measurement process, the house owners should complete a questionnaire to gather information about the building materials used, age and shape or design of the house, etc.

After the exposure time has elapsed, the detector needs to be etched using the NaOH solution, to enlarge the alpha tracks and get ready for optical microscope observation of the appropriate times and temperatures. The etched tracks can then be converted into radon concentrations by using a conversion factor as follows (33).

$${\text{C}}_{\text{Rn}}=\text{Cf}\times \frac{\rho_{Tracks}}{46.8}\times \frac{1000}{T}$$

where C_Rn_ is radon concentration in *Bq.m* ^*−3*^, *C*_*f*_ is calibration factor, r_Tracks_ is track density (number of tracks per *cm* ^*2*^), *T* is the exposure time in hours.

### Discussion

To obtain homogeneous and interrelated results as well as to study the effects of climate change on radon concentration, radon concentration measurements were undertaken during different seasons (34). The measurement period was extended for a year and divided into four periods to represent the four seasons: winter (December–February), spring (March–April), summer (June–August), and autumn (September–November); to ensure that it covered all scenarios (35).

Season variation of indoor radon concentrations was measured in buildings with similar designs in Azarqa town, Jordan (23). An indoor radon survey was conducted in 50 dwellings situated in the Sri Ganganagar district of Rajasthan, using a time-integrated passive technique containing LR-115 type II solid state nuclear track detectors exposed for four seasons (29). Similarly, the seasonal variation of indoor radon concentration was conducted in southern Haryana and Western Utter (27).

The minimum, maximum, and average values of radon concentration in the four seasons in those study areas are given in Tables 1–3. As observed in the three tables, interestingly, radon concentration shows a seasonal variation. The three tables show common results in terms of maximum radon concentration, which was recorded during the winter season, in contrast to the minimum concentration found during the summer season. This is due to the fluctuation of environmental factors and the geological characteristics of the building.

**TABLE 1:** Statistical summary of radon concentration during different seasons inside shops in Alzarqa Town (23).

No.	Season	Min. Con. (Bq/m^3^)	Max. Con. (Bq/m^3^)	Mean Con. (Bq/m^3^)	SD. Con. (Bq/m^3^)
1	Winter	34.8	63.7	45.04	4.21
2	Spring	17	27.1	22.04	3.92
3	Summer	10.2	38.7	16.63	3.94
4	Autumn	16.7	113.2	42.57	9.67

**TABLE 2:** Annual indoor radon levels in Karanpur village in the Sri Ganganagar district of Rajasthan (29).

No.	Season	Min. Con. (Bq/m^3^)	Max. Con. (Bq/m^3^)	Mean Con. (Bq/m^3^)	SD. Con. (Bq/m^3^)
1	Winter	114	249	197	48
2	Spring	99	224	153	43
3	Summer	96	163	141	24
4	Autumn	92	185	159	34

**TABLE 3:** Seasonal variation of indoor radon in southern Haryana and Western Utter (27).

No.	Season	Min. Con. (Bq/m^3^)	Max. Con. (Bq/m^3^)	Mean. Con. (Bq/m^3^)
1	Winter	40.7	80.6	65.2
2	Spring	19.8	29.7	23.5
3	Summer	31.2	54.2	40.2
4	Autumn	22.3	46.8	32.6

*Min. Con., minimum concentration; max. Con., maximum concentration; Mean Con, mean concentration; and SD Con., standard deviation concentration.

The radon concentration in houses with and without a basement was also studied. The presence and absence of the basement affected the variation of radon concentration in buildings. As seen in Table 4, a higher radon concentration was measured in houses with a basement (28).

**TABLE 4:** Radon distribution in houses with and without basements (28).

	Min. Con. (Bq/m^3^)	Max. Con. (Bq/m^3^)	AM (Bq/m^3^)	GM (Bq/m^3^)	M (Bq/m^3^)
House with basement	9	1481	65	39	34
House without basement	28	210	78	58	65

## Conclusion

Radiation is always around us. We are surrounded by natural and manmade radiation sources but long-term radiation exposure can cause adverse health hazards to humans. Radon, which is the sixth radioactive decay products of uranium, contributes almost half of the natural radiation to which human beings are exposed. The International Agency for Research on Cancer, therefore, classified radon as a human carcinogen. Due to its health hazards, different research has been conducted to determine its concentration in a place where human beings live. Indoor radon concentration has a seasonal variation. Its concentration was found to be maximum in the cool (winter) season and a minimum concentration was recorded in the warm (summer) season. The seasonal concentration of indoor radon concentration in homes, offices, schools, hospitals, shops, and industrial buildings needs to be studied further.
